# Visualization of Early RNA Replication Kinetics of SARS-CoV-2 by Using Single Molecule RNA-FISH Combined with Immunofluorescence

**DOI:** 10.3390/v16020262

**Published:** 2024-02-07

**Authors:** Rajiv Pathak, Carolina Eliscovich, Ignacio Mena, Anastasija Cupic, Magdalena Rutkowska, Kartik Chandran, Rohit K. Jangra, Adolfo García-Sastre, Robert H. Singer, Ganjam V. Kalpana

**Affiliations:** 1 Department of Genetics, Albert Einstein College of Medicine, Bronx, NY 10461, USA; rajiv.pathak@einsteinmed.edu; 2 Department of Microbiology and Immunology, Albert Einstein College of Medicine, Bronx, NY 10461, USA; kartik.chandran@einsteinmed.edu (K.C.); rohit.jangra@lsuhs.edu (R.K.J.); 3 Department of Medicine (Hepatology), Albert Einstein College of Medicine, Bronx, NY 10461, USA; carolina.eliscovich@einsteinmed.edu; 4 Department of Developmental and Molecular Biology, Albert Einstein College of Medicine, Bronx, NY 10461, USA; 5 Department of Microbiology, Icahn School of Medicine at Mount Sinai, New York, NY 10029, USA; imena@scripps.edu (I.M.); anastasija.cupic@icahn.mssm.edu (A.C.); magdalena.rutkowska@icahn.mssm.edu (M.R.); adolfo.garcia-sastre@mssm.edu (A.G.-S.); 6 Global Health and Emerging Pathogens Institute, Icahn School of Medicine at Mount Sinai, New York, NY 10029, USA; 7 Department of Medicine, Division of Infectious Diseases, Icahn School of Medicine at Mount Sinai, New York, NY 10029, USA; 8 The Tisch Cancer Institute, Icahn School of Medicine at Mount Sinai, New York, NY 10029, USA; 9 Department of Pathology, Molecular and Cell-Based Medicine, Icahn School of Medicine at Mount Sinai, New York, NY 10029, USA; 10 Departments of Cell Biology, Albert Einstein College of Medicine, Bronx, NY 10461, USA; robert.singer@einsteinmed.edu; 11 Departments of Neuroscience, Albert Einstein College of Medicine, Bronx, NY 10461, USA

**Keywords:** SARS-CoV-2, COVID-19, single molecule RNA-fluorescence in situ hybridization (smRNA-FISH), Immunofluorescence (IF), genomic and sub-genomic RNA replication, spike, nsp12, replication organelles (ROs), nsp3, variants of concern (VOCs)

## Abstract

SARS-CoV-2 infection remains a global burden. Despite intensive research, the mechanism and dynamics of early viral replication are not completely understood, such as the kinetics of the formation of genomic RNA (gRNA), sub-genomic RNA (sgRNA), and replication centers/organelles (ROs). We employed single-molecule RNA-fluorescence in situ hybridization (smRNA-FISH) to simultaneously detect viral gRNA and sgRNA and immunofluorescence to detect nsp3 protein, a marker for the formation of RO, and carried out a time-course analysis. We found that single molecules of gRNA are visible within the cytoplasm at 30 min post infection (p.i.). Starting from 2 h p.i., most of the viral RNA existed in clusters/speckles, some of which were surrounded by single molecules of sgRNA. These speckles associated with nsp3 protein starting at 3 h p.i., indicating that these were precursors to ROs. Furthermore, RNA replication was asynchronous, as cells with RNA at all stages of replication were found at any given time point. Our probes detected the SARS-CoV-2 variants of concern, and also suggested that the BA.1 strain exhibited a slower rate of replication kinetics than the WA1 strain. Our results provide insights into the kinetics of SARS-CoV-2 early post-entry events, which will facilitate identification of new therapeutic targets for early-stage replication to combat COVID-19.

## 1. Introduction

Coronavirus disease 2019 (COVID-19) is a viral respiratory disease that emerged at the end of 2019 in Wuhan, China, and rapidly extended its devastating effects worldwide. COVID-19 is caused by SARS-CoV-2, a large, enveloped, positive-strand RNA virus with a genome approximately 30 kb in length, belonging to the genus *Betacoronavirus* of the family Coronaviridae [1]. As of 27 September 2023, SARS-CoV-2 has continued to spread worldwide, with more than 770,875,433 total confirmed cases and 6,959,316 deaths worldwide [2].

SARS-CoV-2 primarily targets the respiratory tract, and the infection begins when the viral spike protein on the surface of the virus binds to the human receptor angiotensin-converting enzyme (ACE2). Cleavage of the spike protein by the transmembrane protease serine 2 (TMPRSS2) on the surface of epithelial cells triggers the fusion of the viral and host cell membranes, facilitating the entry of the virus into the host cell [3,4]. In addition to causing acute respiratory distress syndrome, SARS-CoV-2 has other pulmonary and extrapulmonary manifestations in the gastrointestinal tract, hepatobiliary system, and cardiovascular, neurological, and renal systems, which often lead to multiorgan failure and shock in severe cases [5,6]. Furthermore, some survivors experience long COVID or post-acute sequelae of SARS-CoV-2 (PASC), with cardiovascular, neurological, and pulmonary manifestations [7,8,9,10]. The exact pathogenesis of acute and chronic disease in extrapulmonary organs in COVID-19 is unknown. It has been suggested that indirect mechanisms such as comorbidities and/or other pathophysiological conditions may play a role [11,12]. Understanding the details of the mechanism of replication and cell tropism of SARS-CoV-2 may provide insights into the pathogenesis and tissue tropism of this virus.

SARS-CoV-2 belongs to coronaviridae, which include enveloped, positive-sense, single-stranded RNA viruses. These viruses employ an elaborate mechanism for replicating their genome and for transcribing the coding sequences. The replication cycle of SARS-CoV-2, like other coronaviruses, begins with the entry of the virus into the cell and release of viral RNA into the cytoplasm [13]. Within the cytoplasm, the first step is the translation of two large open reading frames (ORFs; ORF1a and ORF1b) present at the 5′ end of the positive-strand gRNA for the expression of polyproteins and proteolytic cleavage of these proteins to form 15–16 nonstructural proteins (nsp), of which 15 compose the viral replication and transcription complex (RTC) including RNA-processing and RNA-modifying enzymes [14]. RTC leads to the generation of negative-strand viral RNA replication intermediates from the positive-strand genomic RNA. Discontinuous transcription of the newly synthesized negative-strand RNAs from the 3′ end leads to the formation of a series of shorter sub-genomic RNAs (sgRNAs), which encode for the structural and accessory proteins [1]. During its replication, the virus modifies the intracellular host endoplasmic reticulum membrane to generate the replication organelles (ROs), which are the powerhouses consisting of double-membrane vesicles (DMVs) enclosing the viral RNAs [15,16]. DMVs are likely to provide a protective environment for the replication of gRNA and sgRNA. Newly synthesized gRNA and sgRNA are thought to translocate from the lumen of the DMVs into the cytoplasm through pores present on DMVs. Although the exact composition of the DMVs and pores is not completely understood, it has been established that nsp3 and nsp4 proteins are required for the formation of DMVs and for the biogenesis of DMVs [17].

Many questions remain unanswered about the early replication events of SARS-CoV-2, such as (i) the time it takes for the viral RNA to complete translation and start the replication process after entry into the cytoplasm; (ii) the generation, subcellular localization, and function of the ROs; (iii) the timing of the formation of gRNA, sgRNAs, and RO; and (iv) the mechanism of vRNA synthesis within ROs [13]. Addressing these questions is important not only for obtaining insight into the mechanism of SARS-CoV-2 replication but also for the identification of unexplored drug targets that can be used to curb viral replication at a very early stage of the infection cycle.

Most of the studies aimed at understanding SARS-CoV-2 replication have generally focused on time points ~4–5 h post infection (p.i.), which is when viral replication is at its midpoint, making it easier for monitoring and visualization of the RNA and ROs [18,19]. However, understanding the replication at time points earlier than 4–5 h p.i. may be required to investigate the initial stages of vRNA replication and the formation of ROs. Studying early time points requires highly sensitive and specific methods to detect SARS-CoV-2 RNA at the single-molecule level, as large amounts of RNA are unlikely to be present at these stages. Several reports have utilized single-molecule RNA-FISH (smRNA-FISH) to detect an absolute number of SARS-CoV-2 transcripts [18,20]. However, these reports do not investigate the DMVs at early time points before 4–5 p.i.

To detect and visualize SARS-CoV-2 gRNA and sgRNAs with high specificity and sensitivity at early time points, we employed a combination of single-molecule RNA-fluorescence in situ hybridization (smRNA-FISH) using probes for gRNA and sgRNA and immunofluorescence using nsp3. To facilitate the study of replication kinetics of single SARS-CoV-2 RNA molecules after the virus enters a cell, we infected the cells with a low m.o.i. of virus. Furthermore, we employed high-speed high-resolution scanning fluorescence microscopy (HSHRS-FM) to scan and visualize the replication of SARS-CoV-2 in a large number of cells to determine the different stages of replication in these cells by using smRNA-FISH analysis. The HSHRS-FM method involves scanning the entire slide to create a single high-resolution digital image by tiling and stitching many high-magnification fields of view together, thus capturing the images of a large number of cells present on the slide. We designed probes to simultaneously detect positive strands of the gRNA and sgRNA and carried out a time-course analysis to visualize the cells at time points from 0.5 h to 24 h p.i. Our analyses led to the detection of SARS-CoV-2 gRNA within infected cells as early as 30 min p.i. Subsequent time points indicated that the replicating RNAs were present in distinct spots that contained both gRNA and sgRNAs. We also tested for the formation of DMVs by monitoring nsp3 protein by combining smRNA-FISH with immunofluorescence. Our finding suggests that, at least at the start of the replication (~3 h p.i.), many of the RNA spots were devoid of nsp3 protein, suggesting that some of these RNA spots may represent the direct accumulation of RNA in the cytoplasm without DMV. At later time points, most of the RNA spots became positive for nsp3, suggesting the formation of DMV. We also observed that the replication was asynchronous and cells with various stages of SARS-CoV-2 RNA replication could be found at any given time point. Our studies indicate that combining smRNA-FISH with IF and the utilization of HSHRS-FM enables not only the sensitive detection of SARS-CoV-2 RNAs in a large number of infected cells but also facilitates studies addressing important questions related to the mechanism of early replication events of SARS-CoV-2.

## 2. Materials and Methods

### 2.1. Cell Culture

Vero E6 cells (ATCC, CRL-1586) were maintained in Dulbecco’s Modified Eagle Medium (Cytiva HyClone^TM^, Marlborough, MA, USA; Cat # SH30081.01) supplemented with 10% fetal bovine serum (Atlas Biologicals, Fort Collins, CO, USA; Cat # F-0500-A), 2 mM L-glutamine (Gibco/Thermo Fisher Scientific, Waltham, MA, USA; Cat # 25030-081), non-essential amino acids, 100 U/mL penicillin, and 100 μg/mL streptomycin (Gibco/Thermo Fisher Scientific, Waltham, MA, USA; Cat # 15140-122). All cell lines were maintained in standard 5% CO_2_ in a culture incubator at 37 °C.

### 2.2. Infection of Vero E6 Cells with SARS-CoV-2

For the infection of Vero E6 cells with SARS-CoV-2 (SARS-CoV-2 USA-WA1/2020), cells were seeded on Nunc™ Lab-Tek™ sterile 4-chambered slides (Thermo Scientific™/Thermo Fisher Scientific, Waltham, MA, USA; Cat # 177399) at a confluency of 50–70% the day before infection. On the day of infection, the medium was replaced with 500 μL of infection medium (DMEM supplemented with 2% fetal bovine serum, non-essential amino acids, HEPES, and penicillin/streptomycin) and the cells were transferred to the BSL-3 laboratory. The virus was diluted in infection medium and 100 µL were added to each well to achieve a m.o.I. of 0.5 PFU/cell. After 0, 0.5, 1, 2, 3, 4, 5, 6, 12, and/or 24 h post infection, supernatant was removed and the cells were washed twice with 1 mL of 1× PBS with 5 mM MgCl_2_. After the second wash, cells were fixed with 4% paraformaldehyde. During fixation, cells were protected from light.

### 2.3. Immunofluorescence

The fixed cells were washed with 1× PBS, permeabilized with 0.2% Triton X-100, and incubated with a mouse monoclonal antibody that recognizes the NP protein of SARS-CoV-2 (1C7, kindly provided by Dr. Thomas Moran, Icahn School of Medicine at Mount Sinai). Next, cells were washed with 1× PBS and incubated with Alexa fluor 488 conjugated anti-mouse secondary antibody (Invitrogen/Thermo Fisher Scientific, Waltham, MA, USA) and DAPI. The signals were detected using an EVOS M5000 fluorescent microscope.

### 2.4. smRNA-FISH Probe Design and Specificity Analysis

Forty different 22-nucleotide-long smRNA-FISH probes (5′ → 3′) for spike (S) and RdRp (nsp12) genes were generated using LGC Biosearch Technologies (Petaluma, CA, USA) Stellaris^®^ RNA FISH Probe Designer version 4.2 [21]. Each probe for the spike gene was tagged with Quasar 570 and for the nsp12 gene with Quasar 670 dyes at 3′ ends, respectively. As a target reference sequence, coding sequences (CDSs) of the spike and nsp12 regions were selected from the SARS-CoV-2 Wuhan-Hu-1 (NC_045512.2) reference sequence. Each probe sequence was subjected to BLAST and was screened against other coronavirus sequences, the human transcriptome, and the human intron database. To perform in silico probe sequence specificity analysis, all 40 oligonucleotide sequences of the spike and nsp12 smRNA-FISH probes were aligned against SARS-CoV-2 (NC_045512.2), SARS-CoV-1 Tor2 (NC_004718.3), MERS-CoV isolate HCoV-EMC/2012 (NC_019843.3), HCoV-HKU1 (NC_006577.2), HCoV-OC43 strain ATCC VR-759 (AY585228.1), HCoV 229E strain 229E/human/USA/933-40/1993 (KF514433.1), HCoV NL63 strain NL63/human/USA/0111-25/2001 (KF530112.1), and Human hg38_mRNA (AF001540.1) RefSeq genome or transcriptome assembly using bowtie2 version 2.4.4 [18]. On the other hand, various variants of concern (VOCs) of SARS-CoV-2 [22], in addition to the WA1 strain, were also considered for in silico probe sequence specificity analysis for both spike and nsp12 smRNA-FISH probes. For this analysis, the genomes of several SARS-CoV-2 VOCs, including Alpha/B.1.1.7 (OW998408.1), Beta/B.1.351 (OX008586.1), Gamma/P.1 (MZ427312.1), Delta/B.1.617.2 (OX014251.1), Omicron/B.1.1.529 (OW996240.1), and four sub-variants of Omicron, such as BA.1 (OP810428.1), BA.2 (OM617939.1), BA.4 (OP093374.1), and BA.5 (OP093373.1), along with SARS-CoV-2 Wuhan-Hu-1 (NC_045512.2), were selected, and the alignment was performed using bowtie2 version 2.5.1. To ascertain the minimum edit distance of the oligonucleotide sequences to target the genome/transcriptome, the following bowtie2 arguments were used: score-min L, -0.6, -3 end-to-end -N 0 -L 5 -i S,1,1.15 -D 15 -R 2 -x ${PATH_TO_INDEXED_GENOME} -U ${FASTQ} -S ${OUTPUT_SAMFILE}. Furthermore, to determine the specificity of the designed spike and nsp12 smRNA-FISH probes, the minimum edit distance of the oligonucleotide sequences was also calculated for the spike gene of SARS-CoV-2 and the codon-optimized spike gene of SARS-CoV-2 using bowtie2 version 2.5.1. The 40 different smRNA-FISH probe sequences of the spike and nsp12 genes are shown as Table 1. The heatmaps were created using R v4.0.2 with the Bioconductor package Complex Heatmap v2.9.3 [23].

### 2.5. smRNA-FISH Analysis

Vero E6 cells (ATCC, CRL-1586) were seeded on a four-chambered slide and inoculated with SARS-CoV-2 (USA-WA1/2020) at an m.o.i. of 0.5 for various time points (0, 0.5, 1, 2, 3, 4, 5, 6,12, and/or 24 h). In the case of VOC infection, Omicron BA.1 was used to infect the Vero cells expressing high levels of transmembrane serine protease 2 (TMPRSS2) under similar conditions (at 3, 6, and 12 h). However, Vero E6 cells mock-infected or infected with heat-inactivated SARS-CoV-2 WA1 or SARS-CoV-2 BA.1 at 12 h post infection were considered negative controls. The cells were fixed with 4% paraformaldehyde post infection for 30 min, followed by smRNA-FISH analysis. After fixation, each well of the four-chambered slide was treated with 0.1 M Glycine/PBSM for 10 min at room temperature followed by cell permeabilization with PBSM/0.1% Triton X-100 for 10 min. After washing with 1× PBSM, cells were pre-hybridized in 2× sodium chloride–sodium citrate (SSC) buffer (G-Biosciences, St. Louis, MO, USA; Cat # R019) and 15% formamide (Acros Organics, Geel, Antwerp, Belgium; Cat # 75-12-7) for 30 min at 37 °C. The cells were incubated overnight with 300 μL hybridization buffer containing SARS-CoV-2 spike and/or RdRp/nsp12 stellaris probes tagged with Quasar 570 and Quasar 670 dyes, respectively. SARS-CoV-2 spike and RdRp/nsp12 stellaris probes (250 nM final concentration, LGC Biosearch Technologies, Petaluma, CA, USA) were added to hybridization buffer containing 10% dextran sulfate (Sigma-Aldrich, St. Louis, MO, USA; Cat # D61001), 1 mg/mL competitor tRNA from *E. coli* MRE 600 (Cat No # 10109541001; MilliporeSigma, Milwaukee, WI, USA), 15% formamide (Cat # 75-12-7; Acros Organics, Geel, Antwerp, Belgium), 0.2 mg/mL UltraPure BSA (Ambion™/Thermo Fisher Scientific, Waltham, MA, USA; Cat #: AM2616), 2× sodium chloride–sodium citrate (SSC) buffer (Cat # R019, G-Biosciences, St. Louis, MO, USA), 2 mM ribonucleoside vanadyl complex (Cat # S1402S; New England Biolabs, Ipswich, MA, USA), and 10 U/mL SUPERase•In RNase Inhibitor (Cat # AM2694; Ambion™/Thermo Fisher Scientific, Waltham, MA, USA). When combining RNA-FISH with IF, α-rabbit SARS-CoV-2 nsp3 primary antibody (1:500 dilution, GeneTex, Irvine, CA, USA; Cat # GTX135589) were added to the hybridization buffer. The slides were incubated in a humid hybridization chamber overnight at 37 °C. The next day post incubation, samples were washed with prehybridization buffer (2× SSC, 15% formamide) three times and if they were combined with IF, the slides were incubated two times for 20 min each with anti-rabbit Alexa Fluor 488 secondary Ab (1:1000 in the pre-hybridization buffer, Invitrogen/Thermo Fisher Scientific, Waltham, MA, USA; Cat # A-11008). Following incubation with the secondary antibody, cells were washed with pre-hybridization buffer (2× SSC, 15% formamide) three times followed by 3–4 times washing with 2× SSC. The cells were stained with DAPI (1.0 μg/mL in 2× SSC) for 2 min at room temperature. After DAPI staining, the cells were washed with 2× SSC and mounted using the ProLong Gold antifade mountant (Cat # P10144, Thermo Scientific, Waltham, MA, USA).

### 2.6. Whole-Slide Scanning, Microscope Setup, and Image Acquisition

Fluorescent scanning of the slides was carried out in two stages. First, whole-slide scanning of the entire slide was obtained at 20× using a PANNORAMIC 250 Flash III Slide Scanner. Automatic focusing (default factory settings) was used to scan the whole slide. For fluorescence imaging, we used DAPI, Cy5-Q, and TRITC-Dendra fluorescence filters. Pannoramic scanner software was used for image acquisition and the scanned slides were visualized using CaseViewer 2.4 (64-bit version). To set the parameters for image acquisition, a mock control was used to set the background level of the fluorescence in the test images. After scanning, the same slides were subjected to single-cell imaging. Cells were imaged using an upright, wide-field Olympus BX-63 Microscope equipped with a SuperApochromatic 60×/1.35 NA Olympus Objective (UPLSAPO60XO), a SOLA light engine (Lumencor, Beaverton, OR, USA), an ORCA-R2 Digital Interline CCD Camera (C10600-10B; Hamamatsu, Bridgewater, NJ, USA), and zero-pixel shift filter sets: DAPI-5060C-Zero, Cy3-4040C-Zero (for Quasar 570 detection), and Cy5-4040C-Zero (for Quasar 670 detection) from Semrock, as described [24]. The resulting image pixel dimension was 107.5 nm, and the z-step size (along the optical axis) used for all optical sectioning acquisition was 200 nm. Metamorph software (Molecular Devices, San Jose, CA, USA) was used to control microscope automation and image acquisition. Images were analyzed using ImageJ version 1.54h and/or Fiji software version 2.9.0 [25].

## 3. Results

### 3.1. Design and Optimization of smRNA-FISH Probes to Study the Early Replication Events of SARS-CoV-2 at Single-Cell and Single-Molecule Resolution

**Figure 1:**
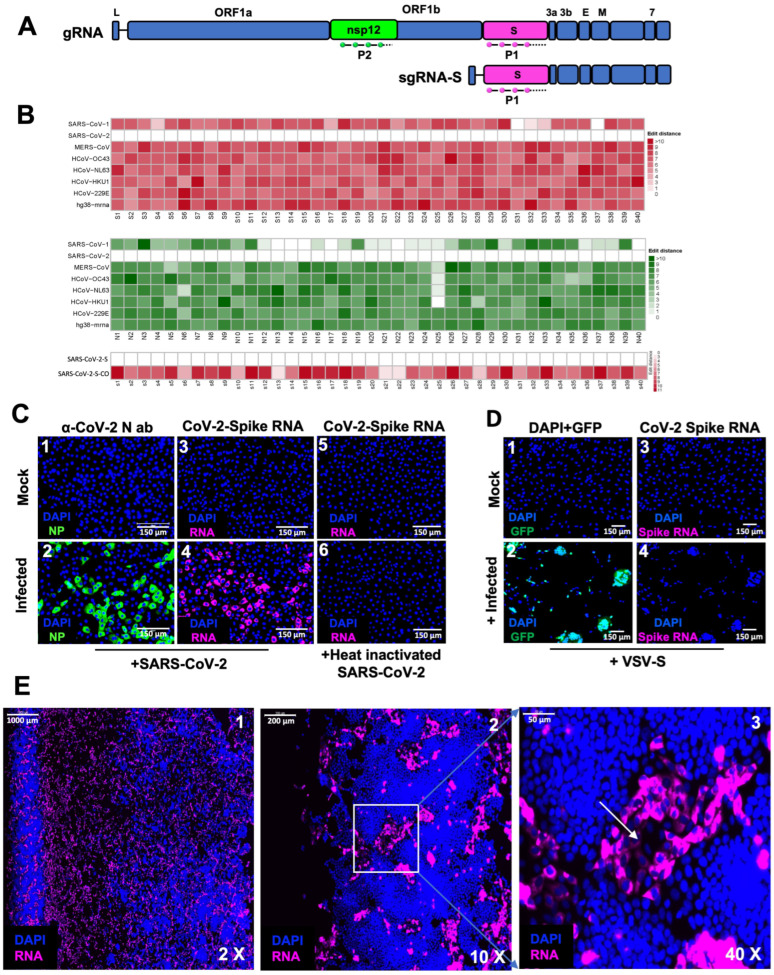
Design and optimization of SARS-CoV-2-specific smRNA-FISH probes to detect SARS-CoV-2 infection. (**A**) Schematic representation of SARS-CoV-2 gRNA and sgRNA-S, indicating the position of the smRNA-FISH probes P1 (in magenta) and P2 (in green) that hybridize to spike and nsp12 ORF sequences, respectively. (**B**) Heatmap representing the probe sequence alignment against various coronavirus genomes (including SARS-CoV-1, SARS-CoV-2, MERS-CoV, HCoV-OC43, HCoV-NL63, and HCoV-229E) and human transcriptomes (hg38-mrna). Each column represents individual 22 nt spike gene probe sequences from the spike gene (S1–S40, upper panel, represented in the shades of red) and from the nsp12 gene (N1–N40, middle panel, represented in shades of green). The lower panel represents the heatmap of the spike probe sequence alignment against the spike gene of SARS-CoV-2 and the codon-optimized spike gene of SARS-CoV-2 (S1–S40, lower panel, represented in the shades of red), showing the specificity of the probe. The minimum edit distance represents the mismatch score between the SARS-CoV-2 sequence and the other genome sequences, where 0 indicates a perfect match (in white) and > 10 represents the most mismatch (in dark red or green). (**C**) Specificity of SARS-CoV-2 spike RNA probe P1 in detecting the SARS-CoV-2 viral genome. Mock- or SARS-CoV-2-infected Vero E6 cells at 12 h p.i. (m.o.i.: 0.5 PFU/cell) were probed with α-NP antibody (green, panels 1 and 2) as a positive control for infection and spike RNA probe P1 (magenta, panels 3 and 4), respectively, to test the specificity of the probe. Panels 5 and 6 shows the specificity of spike RNA probe P1 against Vero E6 cells mock-infected or infected with heat-inactivated SARS-CoV-2 at 12 h p.i. (magenta, panels 3 and 4), respectively. The blue color indicates nuclei upon DAPI staining. Scale bar, 150 µm. (**D**) Vero E6 cells mock-infected or infected with VSV-spike virus containing the codon-optimized spike gene open reading frame were probed with spike RNA probe P1 after 24 h p.i. The cells were imaged under the FITC channel for expression of GFP (shown in green, panels 1 and 2) or under the TritC channel for detecting hybridization with the P1 probe (magenta, panels 3 and 4). GFP expression demonstrates the infection of cells (green, panel 2), whereas the absence of a P1 probe signal (panel 4) with cells infected with VSV-spike indicates the specificity of SARS-CoV-2 spike RNA probe P1. Scale bar, 150 µm. (**E**) Photomicrographs of scanned images of infected cells to determine the extent of SARS-CoV-2 infection and plaque formation. Vero E6 cells infected with SARS-CoV-2 virus at 0.5 m.o.i. were probed with spike RNA probe P1 at 24 h p.i. The entire slide was scanned at a high speed and high resolution. Panels 1–3 represent images at 2×, 10×, and 40× magnification, respectively. The white arrow in panel 3 represents the dead center of a plaque. The blue color represents DAPI staining of nuclei and magenta represents SARS-CoV-2 RNA. Scale bars, 1000 μm, 200 μm, and 50 μm for 2×, 10×, and 40× images, respectively.

To understand the kinetics and spatio-temporal aspects of the SARS-CoV-2 replication at earlier time points and to determine the formation of the timing of membrane-bound ROs, we adopted the method of smRNA-FISH combined with IF to facilitate the sensitive detection of gRNA and sgRNA-S and proteins at the single-cell, single-molecule level. For this purpose, we first optimized the detection of SARS-CoV-2 sgRNA using smRNA-FISH. Forty fluorescently labeled antisense oligonucleotides were synthesized based on the sequence of the spike gene (referred to as the P1 probe), which detected positive strands of gRNA and sgRNA-S (Figure 1A and Table 1; see Section 2.4).

The specificity of the spike oligonucleotide probe set to the SARS-CoV-2 sequence was analyzed by determining the edit distance between the oligonucleotides and the genomic sequences of related human coronaviruses, including SARS-CoV-1, MERS-CoV, HCoV-OC43, HCoV-NL63, HCoV-HKU1, HCoV-229E, and the human transcriptome hg38-mRNA (Figure 1B, top panel). Our analysis indicated that the oligonucleotide probes were specific to SARS-CoV-2 with an edit distance of 0 but not to the other coronaviruses or human sequences with a higher edit distance.

To determine the specificity of the probes for detecting replicating SARS-CoV-2 RNA in cells, Vero E6 cells infected with 0.5 m.o.i. of either infectious virus (SARS-CoV-2 USA-WA1/2020, GenBank MN985325.1) or heat-inactivated virus were subjected to smRNA-FISH using sgRNA-S probe P1 (Figure 1C, panels 3–6). As a positive control, cells were independently analyzed via immunofluorescence (IF) using α-N antibodies to detect viral N protein expression (Figure 1C, panels 1 and 2). RNA-FISH probes and α-N antibodies detected SARS-CoV-2 RNA and N protein, respectively, in infected cells but neither in the uninfected cells nor in the cells infected with heat-inactivated virus, establishing the specificity of the RNA-FISH probes (Figure 1C). As another negative control, we also infected Vero E6 cells with a VSV-spike virus whose genome harbored the codon-optimized spike gene open reading frame (VSV-spike) along with a GFP reporter gene [26]. Since the codon optimization had dramatically altered the coding sequence of the spike gene, we compared the sequences of the 40 different probes with the sequence of the codon-optimized spike gene from VSV-spike and found a much higher edit distance of > 5 between the probes and the target (Figure 1B, lower panel). The VSV-spike virus successfully infected and replicated in the Vero cells, as indicated by GFP expression (Figure 1D, panels 1 and 2). However, we observed that the P1 probe failed to generate a positive signal in these cells infected with VSV-spike (Figure 1D, panels 3 and 4). These results indicated that the P1 probe specifically detected wild-type SARS-CoV-2 Spike RNA sequences and not codon-optimized sequences without any background or false signals (Figure 1D).

Coronaviruses are lytic viruses, and the spreading of infection leads to the formation of plaques that harbor dead cells at the center and newly infected cells at the periphery [27]. To visualize the extent of viral replication, the infected Vero E6 cells were subjected to smRNA-FISH using the P1 probe at 24 h p.i., and the entire slide containing the infected cells was scanned at 20× magnification using HSHRS-FM. We found that at higher magnification, several small plaques characterized by the presence of groups of brightly fluorescent cells positive for the P1 probe surrounding a region of dead cells were visible (Figure 1E). These studies indicated that the P1 probe selectively detected viral RNA in the infected live cells.

### 3.2. Time-Course Analysis of SARS-CoV-2 RNA Replication

**Figure 2:**
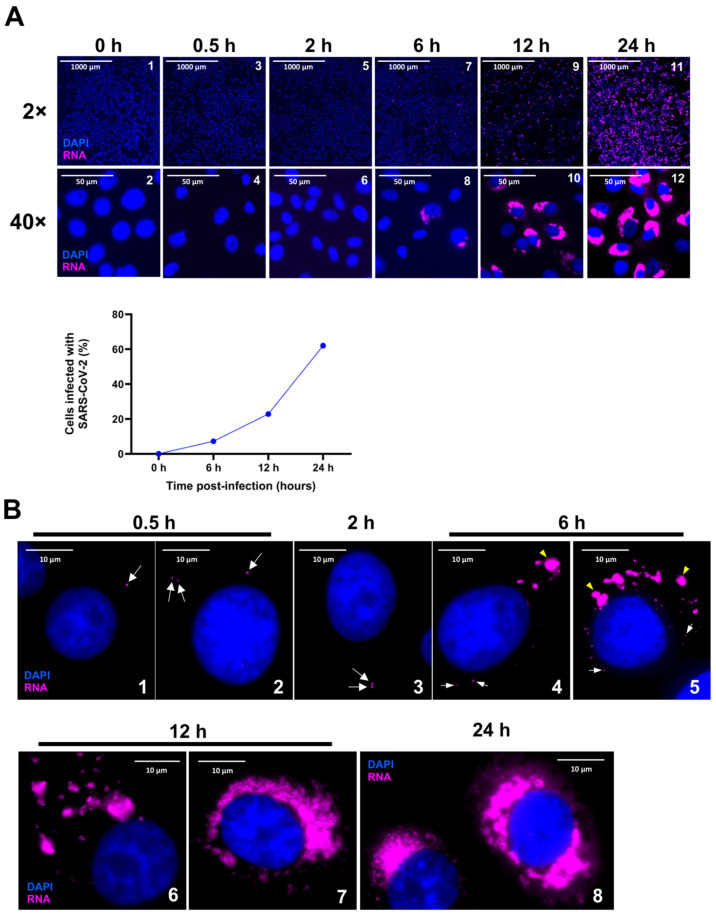
Time-course analysis of SARS-CoV-2 replication using smRNA-FISH. Cells were infected and hybridized with Quasar 570-labeled P1 probes (magenta) against spike RNA at 0, 0.5, 2, 6, 12, and 24 h p.i. (**A**) Photomicrographs representing the images acquired using high-resolution, high-speed scanning microscopy are represented at 2× (upper panels) and 40× (middle panels) magnifications, respectively. Scale bars, 1000 μm and 50 μm for 2× and 40× images, respectively. Graphical representation showing the quantitation of the total percentage of positive cells infected with SARS-CoV-2 at 0, 6, 12, and 24 h p.i. (lower panel). (**B**) Photomicrographs representing images of infected cells at single-molecule resolution probed with P1 at 0.5, 2, 6, 12, and 24 h p.i., acquired using a wide-field Olympus BX-63 microscope. White arrows point to single molecules of SARS-CoV-2 RNA seen at early time points, 0.5 and 2 h p.i. Yellow arrow heads at 6 h p.i. point to RNA clusters/speckles. DAPI (nucleus, blue). Scale bar, 10 μm.

Upon viral entry into the cell, the first step is the synthesis of virally encoded critical RTC enzymes required for replication. The question is how much time it would take to synthesize new RNA molecules from the genomic RNA that entered the cell. To address this question, we carried out a time-course analysis at several time points starting from 0.5 h p.i. earlier than what has been reported [18]. We used 0.5 m.o.i. of the SARS-CoV-2 virus to infect cells, and the entire slide (>100,000 cells) of infected cells was imaged using HSHRS-FM at 20× to determine the percentage of cells infected (Figure 2A). HSHRS-FM allows for a large number of cells on the entire slide to be scanned, but the resolution is not at the single-molecule level, and hence, only cells with a high level of vRNA can be detected using this method. With HSHRS-FM, we observed that at ~6 h p.i., ~7% of the cells were positive for SARS-CoV-2 spike RNA detected by the P1 probe (Figure 2A, panels 7 and 8; see the graph below). At 12 h p.i., ~23% of the cells were positive for RNA, and by 24 h p.i., >50% of the cells were positive, reaching numbers close to the estimated m.o.i. (Figure 2A, panels 9–12; see the graph below). These results suggest that the detectable viral replication can be scored starting from ~6 h p.i. and that at 24 h p.i. viral replication can be easily visualized in >50% of the infected cells when using 0.5 m.o.i. of the virus (Figure 2A, panels 11 and 12).

To visualize SARS-CoV-2 replication at the single-molecule level, the above samples were imaged using a fluorescence microscope fitted with a wide-angle lens and 1.4 numerical aperture (N.A.) with a 60× magnification oil objective that allows for the detection of single viral RNA molecules as diffraction-limited puncta within the infected cells. Since the Stellaris FISH probe comprises 40 tandem 22 mer oligonucleotides corresponding to contiguous sequences on an mRNA, each with a fluorescent label, these probes collectively bind along the same target transcript to produce a punctate signal that appears as bright, diffraction-limited, computationally identifiable fluorescent spots [28,29,30]. Furthermore, the large number of probes in a Stellaris FISH assay ensures a high level of sensitivity and specificity and minimizes false negatives and background signals. We found that cells at 0.5 and 2 h p.i. exhibited single molecules of viral RNA, which were not detected either in the uninfected samples or in the infected samples with heat-inactivated virus (Figure 2B, panels 1–3, white arrows). Furthermore, positive cells at early time points exhibited the presence of one, two, or occasionally three spots of RNA per cell, suggesting that this was due to random infection and possibly multiple viruses entering the cells (Figure 2B, panels 1–3; white arrows). However, we did not observe any cells that had more than three spots of RNA, suggesting that these RNA spots might have represented the earliest viral RNA that entered the cell. Analysis of Z-stacks of images revealed that the RNA signals in these images were detected only in the stacks that corresponded to interior of the cell and not the periphery, suggesting that these spots represented the genomic RNA molecules that had entered the cytoplasm (Figure S1A). Furthermore, we found that some cells exhibited the presence of SARS-CoV-2 RNA within the nucleus at 30 min p.i (Figure S1B). At time points after 6 h p.i., RNA spots that represented single molecules as well as larger patches that represented groups of multiple RNA molecules were visible (Figure 2B, panel 4–5; white arrows and yellow arrowheads). Most of the FISH signals within the cells at 6 h p.i. corresponded to patches of RNA rather than single RNA molecules (Figure 2B, panels 4 and 5; white arrows and yellow arrowheads). By 12 h p.i., the size and number of these patches had increased, and in most of the cells, the entire cytoplasm was filled with patches of viral RNA (Figure 2B, panels 6–7). By 24 h p.i., the majority of the infected cells exhibited viral RNA that filled the entire cytoplasm (Figure 2B, panel 8).

### 3.3. Replication of gRNA and sgRNA during Early Stages of SARS-CoV-2 Viral Replication

**Figure 3:**
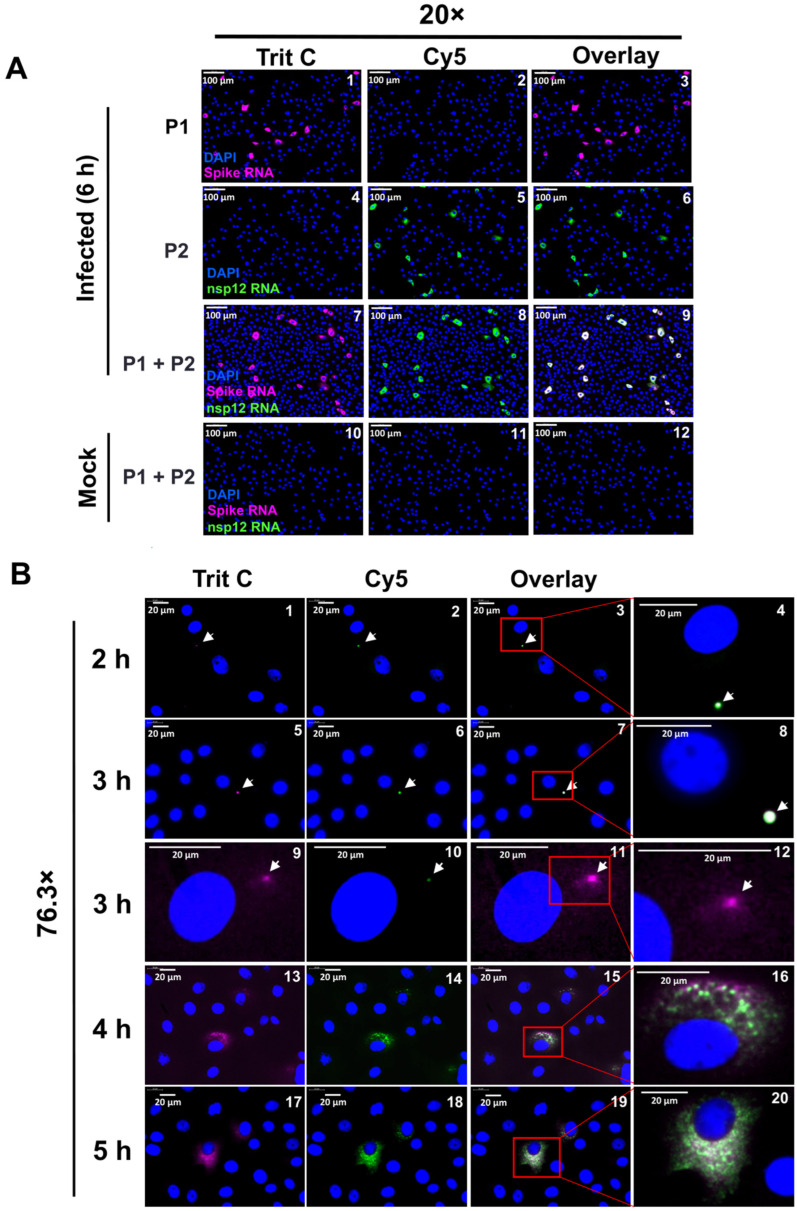
Simultaneous detection of SARS-CoV-2 gRNA and sgRNA-S in a time-course analysis. (**A**) Validation of spike RNA probe P1 and nsp12 RNA probe P2 to simultaneously detect gRNA and sgRNA-S in the infected cells. Cells were infected for 6 h and hybridized with probes P1 and P2 alone or together and subjected to smRNA-FISH. Images are represented at 20× magnification. Scale bar at 100 μm. (**B**) A time-course analysis to detect the replication of gRNA and sgRNA in infected cells: The infected cells at 2, 3, 4, and 5 h p.i. were hybridized with both spike RNA probe P1 and nsp12 RNA probe P2 and subjected to high-speed, high-resolution scanning. The images are represented at 76.3× magnifications. Panels 4, 8, 12, 16, and 20 are the zoomed-in images of insets for the indicated time points. White arrows point to RNA spots that are surrounded by potentially single-molecule sgRNA-S. Blue indicates DAPI-stained nuclei. Magenta indicates sgRNA-S, green indicates gRNA and white color indicates the overlap of the two probes. Scale bar at 20 μm.

Since spike RNA probe P1 detected both gRNA and sgRNA-S, we tested the ability of a second probe set (P2) derived from *nsp12* gene sequences within ORF1b to detect the gRNA alone (Figure 1A and Table 1). The specificity of oligonucleotides to SARS-CoV-2 *nsp12* sequences was analyzed by determining the edit distance between the oligonucleotides and the genome sequences of related human coronaviruses, including SARS-CoV-1, MERS-CoV, HCoV-OC43, HCoV-NL63, HCoV-HKU1, HCoV-229E, and the human transcriptome hg38-mRNA (Figure 1B, middle panel). Our analysis indicated that the oligonucleotide probes were specific to SARS-CoV-2 with an edit distance of 0 but not to the other coronaviruses or human sequences with a higher edit distance. The specificity of P1 and P2 probes were tested by carrying out smRNA-FISH analysis using either P1 or P2 probe sets or both probe sets together, and images were analyzed in both TritC (P1 probe) and Cy5 (P2 probe) channels (Figure 3A). We found that the probes were specific and that there was no bleeding from one channel to the other (Figure 3A, panels 1–6). When the two probes were used together, signals from both probes were detected in the same set of infected cells (Figure 3A, panels 7–9). Neither of the probes detected any signal in mock-infected cells, confirming probe specificity (Figure 3A, panels 10–12).

We carried out another time-course analysis using the two probes together to determine the early replication kinetics of gRNA and sgRNA-S. As before, we used a low m.o.i. of the virus for infection, aiming to track the replication of a single virus shortly after its entry into the cell at time points of 0, 2, 3, 4, 5, and 6 h p.i. Scanning of the entire slides containing the infected cells and analyzing the images at low magnification (5×) indicated the presence of strong positive cells at 4 h p.i but none at 0–3 h p.i. (Figure S2, panels 1–15 and 16–18). The infected cells were positive for both gRNA and sgRNA-S (Figure S2, panels 16–18). As time progressed, the number of positive cells increased at 5 and 6 h p.i. (Figure S2, panels 19–24). We did not observe any positive cells within mock infected samples (Figure S2, panels 25–27). Analysis of the above images at higher magnification (76.3×) indicated that a majority of the infected cells at 2 h p.i. and many cells at 3 h p.i. appeared to have single RNA spots positive for both the P1 and the P2 probe, indicating that these spots represented viral RNA in the process of replication in the early stages (Figure 3B, panels 1–8, white arrows). Because of the resolution limitations of HSHRS-FM, it is likely that the single positive RNA spots we identified were not single RNA molecules but rather represented groups of RNA molecules. At 3 h p.i., some cells harbored single RNA clusters/patches that were larger than those at 2 h p.i. and showed distinct regions of hybridization to the P1 and the P2 probes (Figure 3B, panels 9–12). Within these RNA clusters/spots, there was a central area positive for both P1 and P2 probes, which was surrounded by smaller spots that hybridized only to the P1 probe. This indicated that perhaps the central region of these clusters harbored gRNA and that the RNA molecules in the periphery of this central region corresponded to sgRNA-S that hybridized to the P1 probe only (Figure 3B, panels 9–12). We surmised that the single spots observed in each cell at 2 h and 3 h p.i. likely represented the early stages of RNA replication initiated by the SARS-CoV-2 RNA that entered the cell. At 4 and 5 h p.i., the infected cells harbored multiple viral RNA spots that were visualized as large puncta of varied size and number (Figure 3B, panels 13–20).

**Figure 4:**
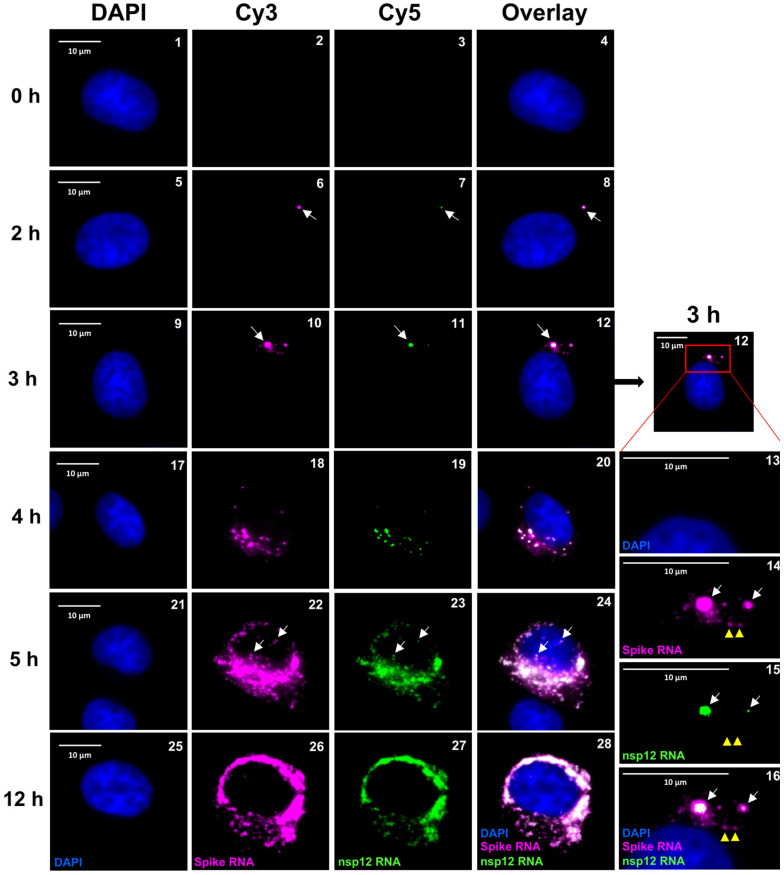
Single-molecule analysis to determine the kinetics of SARS-CoV-2 gRNA and sgRNA replication. Transcript-specific visualization of gRNA and sgRNA-S in SARS-CoV-2-infected Vero E6 cells using the P1 (spike, represented in magenta) and P2 (nsp12, represented in green) probes. When spike RNA probe P1 and nsp12 RNA probe P2 both hybridized to the same molecule, it is shown in white (overlay). Blue indicates DAPI-stained nuclei. Images represent the cells infected with SARS-CoV-2 and hybridized with the P1 and the P2 probes at 0, 2, 3, 4, 5, and 12 h p.i. The white arrows in panels 6–16 point to replication centers. The yellow arrowheads point to single molecules of sgRNA-S. Panels 13–16 show the magnified area of the corresponding overlay images at 3 h p.i., illustrating two replication centers (white arrows). The larger RNA spots in these panels harbor gRNA in the center surrounded by sgRNA-S. Single-molecule RNA are indicated by yellow arrowheads (panels 14–16). The white arrows in panels 22–24 point to RNA in the nucleus. The images were acquired using a wide-field microscope to detect the diffraction-limited single molecules. Scale bar at 10 μm.

**Figure 5:**
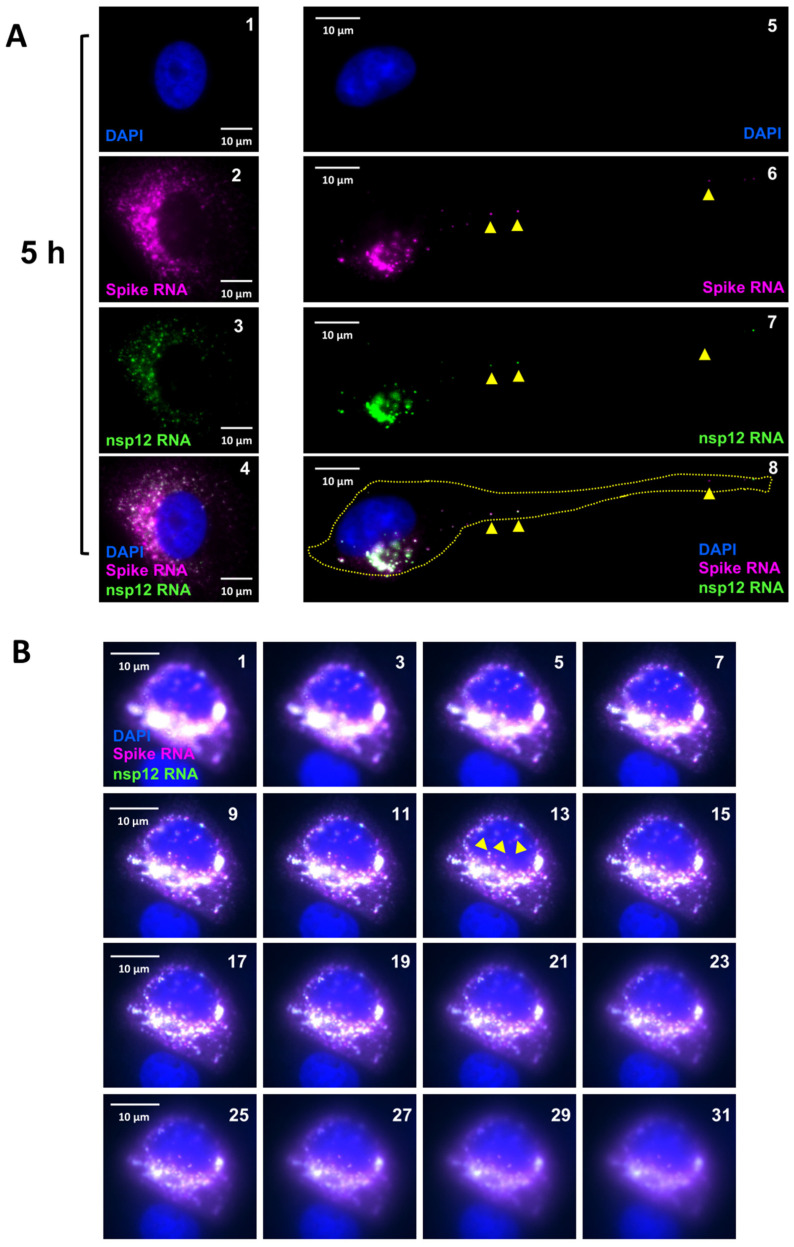
Images of cells infected with SARS-CoV-2 at 5 h p.i. (**A**) Representative cells infected with SARS-CoV-2 at 5 h p.i. Panels 1–4 represent a cell filled with ROs at 5 h p.i. Panels 5–8 represent a single cell with gRNA and sgRNA-S migrating to the distal end of the cell (yellow arrowheads). Yellow dotted line in panel 8 represents the outline of the cell. (**B**) Presence of SARS-CoV-2 RNA in the nucleus. The images from a few Z-stacks of the cell are represented to demonstrate the presence of the positive RNA spot inside the nucleus. The Z-stack images were acquired starting from the bottom of the cell moving upwards. The panel numbers represent the stack number in a total of 41 optical sections (each with a 200 nm Z step size). In both A and B, images were acquired using a wide-field microscope to detect the diffraction-limited single molecules. The spike RNA P1 probe signals are colored in magenta (sgRNA-S), nsp12 RNA P2 probe signals are colored in green (gRNA), and when P1 and P2 both hybridized to the same molecule, it is shown in white (overlay). Scale bar, 10 μm. DAPI (nucleus, blue).

To gain further insight into the positive SARS-CoV-2 RNA spots observed at early time points (2–3 h p.i.), the above samples were subjected to single-molecule microscopy analysis, as described earlier (Figure 4). We found that, at 2 h p.i., many cells harbored either one or two RNA spots that hybridized to both the P1 and the P2 probe, indicating that these were gRNAs (Figure 4, panels 5–8, white arrows). However, the RNA spots hybridized differentially to the P1 and P2 probes. The signal for the P1 probe was more intense and exhibited a larger area than that for the P2 probe, which was restricted to the center of the same spot. Furthermore, we did not detect sgRNAs that were distinct from the gRNA spots at this point (Figure 4, panels 5–8, white arrows). At 3 h p.i., we found an increase in the number and size of spots that had hybridized to both the P1 and the P2 probe (Figure 4, panels 9–16). The size of the spots within the same cell varied considerably, suggesting that these spots represented clusters of RNA at various stages of replication (Figure 4, panels 12 and 16). As we had noticed in the low-resolution analysis, the central areas in these spots hybridized to both the P1 and the P2 probe, representing gRNA, and were surrounded by radiating spots that hybridized to only the P1 probe, representing sgRNA-S (Figure 4, panels 9–16). The size of the peripheral spots around the RNA spots that hybridized to the P1 probe alone were similar in size to that of a single RNA molecule (Figure 4, panels 14–16, white arrows point to central larger RNA spots and yellow arrowheads points to single molecules of sgRNA-S). These results suggested that the central larger RNA spots that hybridized to both the P1 and the P2 probe represented replication centers with genomic RNA, and the single-molecule RNA spots that hybridized to P1 alone around the periphery of these spots represented sgRNA. We observed that the number and size of the RNA spots increased at later time points and that the intensity with which these spots hybridized to the P1 and the P2 probes varied (Figure 4, panels 17–28, and Figure 5, panels 1–8). At later time points of 5–12 h p.i., the RNA spots had spread out from nuclear periphery, filling the entire cytoplasm (Figure 4, panels 21–28, and Figure 5A, panels 1–4). In some cells, we noted that both gRNA and sgRNA were migrating away from the nuclear periphery and had reached the narrow and elongated distal regions of the cells (Figure 5A, panels 5–8, yellow arrowheads).

### 3.4. Nuclear Localization of SARS-CoV-2 RNA

Despite being a surface transmembrane glycoprotein, the S protein in SARS-CoV-2 has a unique nuclear localization signal (NLS) called PRRARSV that is absent in other coronaviruses, suggesting its potential translocation into the cell nucleus. In a recent study by Sattar et al., the presence of SARS-CoV-2 spike (S) protein and spike mRNA was observed within the cell nucleus of SARS-CoV-2-infected airway epithelium, unveiling nuclear co-localization as a novel aspect of SARS-CoV-2 pathogenesis [31]. Interestingly, we also observed both gRNA and sgRNA-S RNA in the nucleus (Figure 4, panels 21–24, white arrows). To ascertain whether these spots were within the nucleus, we examined the Z-stacks (with a step size of 200 nm) taken from the top to the bottom of the cells along the optical axis and found that these RNA signals were indeed present in the stacks that corresponded to the interior of the nucleus (Figure 5B, yellow arrowheads). Nevertheless, it remains unclear whether these nuclear viral RNAs had a substantial impact on viral replication.

### 3.5. Nature of RNA Spots Observed during Early Stages of Viral Replication

**Figure 6:**
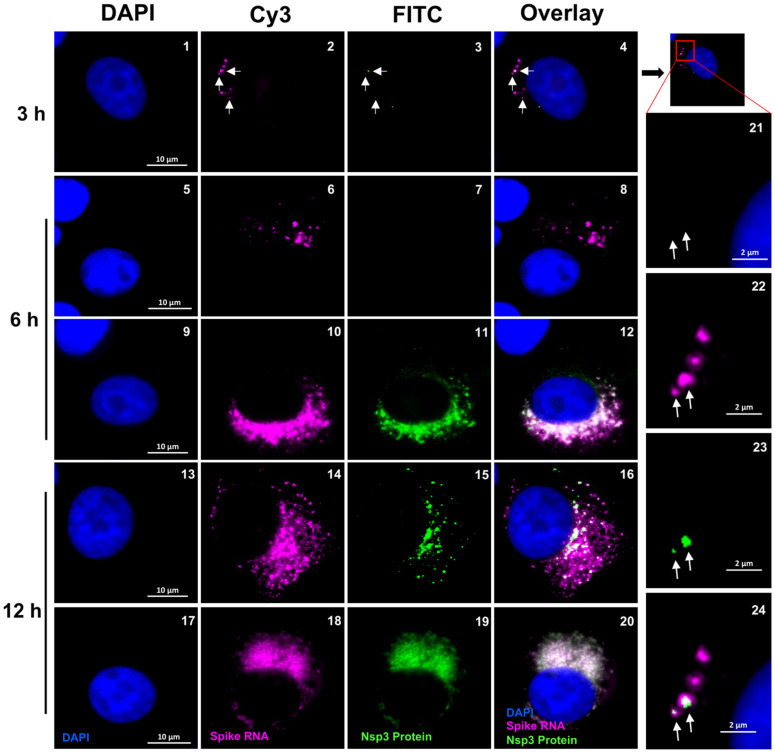
Co-localization of SARS-CoV-2 RNA speckles with nsp3 protein. Representative cells showing the co-localization of spike probe P1 and nsp3 protein within the Vero E6 cells infected with SARS-CoV-2 at 3, 6, and 12 h p.i., respectively. The images were acquired using a wide-field microscope to detect the diffraction-limited single molecules. The blue color represents DAPI nuclear staining, the green color represents nsp3 protein, and the magenta color represents SARS-CoV-2 spike RNA. Scale bar, 10 μm. Panels 21–24 show the magnified area of the corresponding images at 3 h p.i. (scale bar at 2 μm). White arrows indicate RNA speckles that co-localized with nsp3. Black arrow point to the same panel as in panel 4, whose blown up images are shown in panels 21–24.

To determine whether the RNA spots that were larger than single molecules that we observed at time points 3 h and later represented the replication center or organelles (ROs), we employed a combination of RNA-FISH and immunofluorescence using α-nsp3 antibodies. The SARS-CoV-2 nsp3 protein, as previously documented, is associated with double-membrane vesicles (DMVs) that surround the viral RNA, a prominent characteristic of SARS-CoV-2 ROs within the host cell [15,32]. We surmised that colocalization of nsp3 protein with RNA spots could be used to indicate the formation of ROs. To determine whether the RNA spots we observed co-localized with nsp3, we infected cells with SARS-CoV-2 and carried out combined RNA-FISH with IF using the P1 probe to detect sgRNA and α-nsp3 antibodies to detect the protein at three different time points p.i. (Figure 6). Our results indicated that, at 3 h p.i., a few cells were positive for RNA spots. Within these cells, approximately one third of the RNA spots had colocalized with nsp3, whereas the remaining RNA spots were free of nsp3 (Figure 6, panels 1–4 and panels 21–24). Furthermore, nsp3 co-localization was independent of the size of the spots, indicating that the lack of colocalization was not due to the limitation in detecting nsp3 signals in smaller spots (Figure 6, panels 1–4 and panels 21–24). By 6 h p.i., more cells had RNA spots that had colocalized with nsp3 (Figure 6, panels 5–12). Furthermore, there was variability in the number of RNA spots per cell as well as the colocalization of nsp3 with RNA at 6 h p.i. (Figure 6, panels 5–12). Whereas some cells expressed RNA spots but no nsp3 (Figure 6, panels 5–8), other cells expressed both RNA spots and nsp3 protein, and most of the RNA spots had colocalized with nsp3 (Figure 6, panels 9–12). By the 12 h mark, more cells had expressed RNA spots and the majority of the RNA spots had overlapped with nsp3 (Figure 6, panels 13–20). These results suggested that there was heterogeneity in the colocalization of nsp3 with RNA spots at early time points. This heterogeneity did not reflect on the expression of nsp3 in the cells, as within the same cells both kinds of RNA spots—both with and without nsp3—were observed. Based on these results, we hypothesized that although the individual diffraction limited positive spots at 2 h p.i. and earlier time points likely represented single gRNA molecules, the larger RNA spots observed at 3 h p.i. represented clusters of RNA that were likely to be precursors to the formation of RO. Some of these RNA clusters were devoid of nsp3 at early time points. By later points, all the RNA spots had colocalized with nsp3, suggesting the successful formation of DMVs.

### 3.6. Heterogeneity in the Replication of SARS-CoV-2 RNA

**Figure 7:**
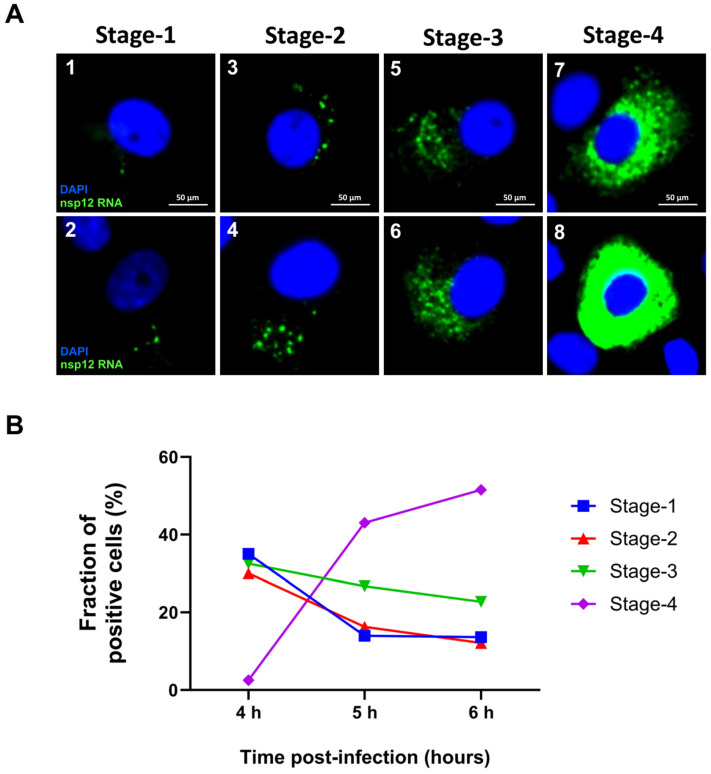
Quantitation of cells containing viral RNA at different stages of replication. (**A**) Examples of images of cells with four different stages of viral replication at stage 1 (panels 1 and 2), stage 2 (panels 3 and 4), stage 3 (panels 5 and 6), and stage 4 (panels 7 and 8). The blue color represents DAPI nuclear staining, and nsp12 RNA (P2 probe) signals are colored in green, which represents replication centers containing genomic RNA. Scale bar, 50 μm. (**B**) Graphical representation of the quantitation of the percentage of positive cells harboring four different stages of replication at 4, 5, and 6 h p.i.

Previous reports had indicated that within the pool of infected Vero E6 cells, about 10% were “super permissive,” allowing a high degree of replication of virus, based on the observation of the density of SARC-CoV-2 RNA spots per individual cells at 8 h p.i. [18]. To further examine this information, we took advantage of the ability of HSHRS-FM to scan a large number of cells on the slides at three different time points p.i. We observed heterogeneity in the replication of the SARS-CoV-2 at any given time point, consistent with the previous report (Supplementary Figure S3). However, we found a random distribution of cells with varying densities of RNA spots at any given time point. At early time points, the majority of infected cells had either one or a few RNA clusters per cell and a few cells had a large number of RNA spots (Supplementary Figure S3, panel 1). At later time points, the majority of infected cells had a large number of RNA clusters filling the entire cytoplasm (Supplementary Figure S3, panels 2 and 3). To quantitate the percentage of cells containing varying densities of RNA spots per cell, we used the images obtained with HSHRS-FM with the P2 probe to quantitate the distribution of cells with differing densities of RNA clusters per cell. We arbitrarily defined various stages of replication as follows: Stage I was when cells harbored one to five RNA spots per cell but not much diffused RNA (Figure 7A, panels 1 and 2). Stage 2 and stage 3 were defined as intermediates, where the number of RNA spots progressively increased (Figure 7A, panels 3–6). Stage 4 was defined as a late stage during which the entire cytoplasm was filled with RNA spots and distinct RNA spots could not be quantified (Figure 7A, panels 7 and 8). We analyzed ~1000 cells per time point at 4, 5, and 6 h p.i. by counting cells in 10 different random fields of view using the images obtained with HSHRS-FM. The percentage of cells at these four stages was determined as the fraction of positive cells that exhibited different densities of RNA spots at a given time point (Figure 7B). The results indicated that, at 4 h p.i., approximately 35% of the infected cells showed viral replication at stages 1–3 and a few cells (2.5%) at stage 4. At 5 h p.i., stages 1–3 had decreased to 14 to 26%, whereas stage 4 had increased to 43%. At 6 h post infection, the majority of cells exhibited stage 4 replication. Although there were very few cells with stage 4 (2.5%) replication at 4 h p.i., at 6 h p.i., 51% of the infected cells were at stage 4 (Figure 7B). These data suggested that the virus replication was asynchronous and heterogeneous.

### 3.7. Specificity of SARS-CoV-2 Probes against Variants of Concern (VOCs) of SARS-CoV-2

**Figure 8:**
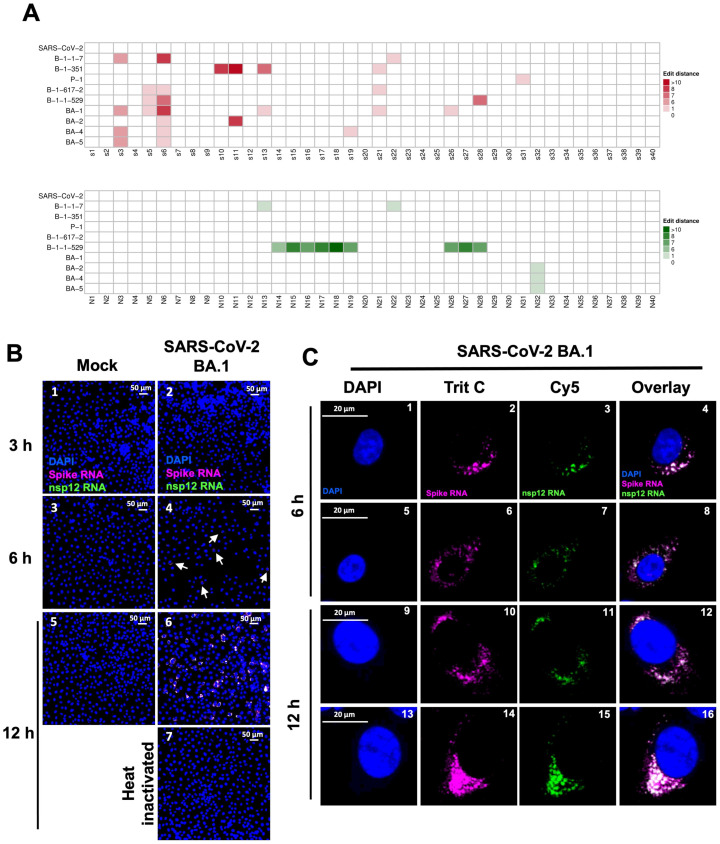
Ability of SARS-CoV-2 probes to detect variants of concern (VOCs) of SARS-CoV-2. (**A**) Heatmap representing the probe sequence alignment against various genomes of SARS-CoV-2 variants of concern (VOCs) (SARS-CoV-2 B.1.1.7, B.1.351, P.1, B.1.617.2, B.1.1.529, BA.1, BA.2, BA.4, and BA.5) along with SARS-CoV-2 Wuhan-Hu-1. Each column represents individual 22 nt spike gene probe sequences from the spike gene (S1–S40, upper panel, represented in the shades of red) and from the nsp12 gene (N1–N40, lower panel, represented in shades of green). The minimum edit distance represents the mismatch score between the SARS-CoV-2 Wuhan-Hu-1 gene sequence and the other SARS-CoV-2 VOC genome sequences, where 0 indicates a perfect match (in white) and > 10 represents the most mismatch (in dark red or green). (**B**,**C**) Validation of spike RNA probe P1 and nsp12 RNA probe P2 to simultaneously detect gRNA and sgRNA-S in the Vero-TMPRSS2 cells infected with SARS-CoV-2 BA.1 VOC. Cells were infected at 3 h, 6 h, and 12 h. (**B**). Panels 2, 4, and 6 indicate infected cells and panels 1, 3, and 5, represent mock-infected cells, shown at a 20× magnification. Panel 7 indicates Vero-TMPRSS2 cells infected with heat-inactivated SARS-CoV-2 BA.1 at 12 h p.i. under similar conditions. Scale bar at 50 μm. White arrows point to infected cells in panel 4. (**C**). Visualization of representative single Vero-TMPRSS2 cells infected with SARS-CoV-2 BA.1 VOCs at 6 h (panels 1–8) and 12 h (panels 9–16) post infection. Scale bar at 20 μm. Magenta = spike RNA probe P1; green = nsp12 RNA probe P2; blue = DAPI staining.

To determine whether the spike (P1) or the nsp12 (P2) probe that we designed using WA1 strain can also detect variants of concern (VOCs) of SARS-CoV-2, we conducted probe sequence alignment against the genomes of several SARS-CoV-2 VOCs, including VOCs Alpha/B.1.1.7, Beta/B.1.351, Gamma/P.1, Delta/B.1.617.2, Omicron/B.1.1.529, and four sub-variants of Omicron (BA.1, BA.2, BA.4, and BA.5), along with SARS-CoV-2 Wuhan-Hu-1 (Figure 8A). This alignment was based on the edit distance, which quantifies the mismatch score between the gene sequence of SARS-CoV-2 WA1 and the genome sequences of other SARS-CoV-2 VOCs, ranging from 0 for a perfect match to >10, indicating a significant mismatch (Figure 8A). The Omicron variants, as the fifth designated VOCs, exhibit an extensive spike protein mutation profile, surpassing more than 30 mutations in the spike regions [22]. They have displayed a remarkable 13-fold increase in viral infectivity and are 2.8 times more contagious than the Delta variants [22]. Consequently, our next objective was to confirm the probe’s specificity against the Omicron variants [22]. Based on the edit score, one of the first identified Omicron variants (BA.1) of SARS-CoV-2 was found to have the maximum number of probe mismatches for spike probe P1. Specifically, we found that six of the oligonucleotides in a mixture of 40 different oligonucleotides of the P1 probe targeted to the spike protein exhibited mismatches, and none of the sequences in the P2 probe, which targets the nsp12 sequence, exhibited mismatches to the sequences of Omicron BA.1 variant (Figure 8A). To determine whether the P1 and P2 probes can detect BA.1 VOC gRNA and sgRNA, Vero cells expressing high levels of transmembrane serine protease 2 (TMPRSS2) were infected with 0.5 m.o.i. of the SARS-CoV-2 BA.1 strain at 3, 6, and 12 h post infection and probed using the P1 and P2 probes (Figure 8B, panels 2, 4, and 6). We found that although the P1 probe hybridized with a slight decrease in intensity, both the P1 and the P2 probe were able to detect the BA.1 VOC and the signals co-localized with each other in the same cells, suggesting that these probe sets can be used for detecting VOC (Figure 8B, panels 2, 4, and 6). It has been reported that Omicron variants exhibit slower replication kinetics at early time points (12 and 24 h p.i.) [33,34,35]. Consistent with these reports, the infectivity of the BA.1 strain was much lower compared to that of the WA1 strain (Figure 8; compare to Figure 2 and Figure 3). However, the signals were clearly above the background, as neither uninfected mock cells nor cells infection with heat-inactivated SARS-CoV-2 BA.1 virus used in the same experiment showed any positive signals under similar conditions (Figure 8B, panels 1, 3, 5, and 7). Examination of cell images at higher magnification clearly indicated that both the P1 and the P2 probe were overlapping and that the RNA was present in patches similar to what we observed with the SARS-CoV-2 WA1 strain, suggesting actively replicating centers of viral RNA (Figure 8C, panels 1–16). These results establish that the P1 and P2 probes can be efficiently used to detect gRNA and sgRNAs of VOCs.

## 4. Discussion

Our studies provide a spatial and temporal characterization of early post-entry events of SARS-CoV-2 RNA replication in Vero E6 cells at a single-cell, single-molecule resolution and shed light on the formation of replication centers at early time points. Our data complement previous reports and provide information and clear images of very early stages of replication. We visualized cytoplasmically localized single gRNA molecules within the cell cytoplasm at 0.5 h p.i., indicating that these molecules likely represented viral RNA from a particle that had just entered the cell. To our knowledge, these represent the earliest time points of SARS-CoV-2 viral RNA detected in an infected cell. Our studies also indicate that, within two hours, single distinct RNA spots/clusters had formed within the cytoplasm that were larger than the diffusion-limited single molecules, suggesting that these are results of the initial replication of a single gRNA that had entered the cell. By 3 h p.i., we found that some but not all of these RNA clusters were associated with nsp3, which is a marker for the formation of ROs that are characterized by the presence of DMVs, suggesting that these RNA clusters/speckles were likely precursors to the formation of RO. Eventually, most of the RNA speckles were associated with nsp3 protein at later time points. This temporal association of nsp3 with RNA clusters strongly supports the notion that RNA clusters/speckles that are formed without nsp3 might serve as precursors to ROs during the early phases of SARS-CoV-2 replication. It is interesting to note that the earliest time of reported co-localization of nsp3 with viral RNA, in a previous study conducted by Shi et al., was 6 h p.i. In that study, the intracellular localization and timing of expression of the viral nsp3 protein in SARS-CoV-2-infected cells were investigated at multiple time points (2, 3, 4, 6, 8, and 24 h p.i.), with the first appearance observed at 6 h p.i. [36]. In our study, we were able to distinctly visualize the presence and co-localization of nsp3 in some of the RNA clusters at 3 h p.i., possibly attributed to the heightened sensitivity of our RNA-FISH approach. It is intriguing to note that many of the RNA clusters at the 3 h time point were without nsp3. It is not clear whether these RNA spots without nsp3 were aggregates or phase-separated RNA clusters in the cytoplasm without a membrane. More research is needed to characterize these RNA spots that lacked nsp3. It is also intriguing to note that, although gRNA and nsp3 appeared to reside in the center of the RNA clusters, the sgRNA appeared to surround these centralized structures. One possibility is that if the RNA clusters represented RO, then sgRNA at the periphery of these clusters may have represented the those that had migrated out of the ROs.

Our studies also indicate that there was cell-to-cell heterogeneity and asynchrony in the rate of RNA replication, which could be observed by quantitating the number and nature of RNA spots/clusters present within cells at a given time point. When infected with low m.o.i. of 0.5, most of the cells had one or a few RNA spots/clusters that appeared to be at an early stage of replication. As time progressed, the percentages of cells with early stages of RNA replication decreased, and those with late stages of replication increased. Thus, the heterogeneity appeared to be a random or stochastic event, as all stages of replication were found at any given time point. The reason for the heterogeneity in replication in different cells was not clear and could have been due to differences in the rate of entry of RNA into the cytoplasm, the establishment of the first RO from the RNA that entered the cell, or differences in the rate at which the RNA replication progressed within the cells. It is well known that the stage of the cell cycle and other cellular factors affect the rate of viral replication [37,38].

Consistent with previous observations [31], we also identified SARS-CoV-2 gRNA molecules within the nuclei of certain infected cells. Recent evidence has stirred controversy in the field, suggesting that, at least under certain specific conditions, SARS-CoV-2 sequences can undergo retrotranscription and integrate into the host genome as DNA, leading to the formation of chimeric genes [31,39,40]. At this point, we do not have any information regarding the integration of SARS-CoV-2 nucleic acid into the nucleus, but we clearly found both gRNA and sgRNA within the nucleus. Interestingly, we did not find nsp3 protein in the nucleus, suggesting that the replication centers are limited to the cytoplasm. More experiments are needed to establish the role of the viral components in the nucleus during replication of the virus.

In summary, high-resolution early kinetic analysis of SARS-CoV-2 RNA replication provides an understanding of the timing of the formation and arrangement of gRNA and sgRNA and possibly the formation of RO, and it sets the stage for further analysis of these unique organelles in the future. While our manuscript was in preparation, several groups reported on the use of smFISH to study the infection of SARS-CoV-2 in human cell lines [18,20,41,42]. Although our data are consistent with these studies, we were able to identify viral RNA within the cells at earlier time points (0.5 h and 1 h p.i.) and were able to determine that the RNA spots could have been precursors to ROs or viral RNA factories at a much higher resolution.We identified the presence of a central region within these ROs filled with gRNAs and sgRNAs that were surrounded by migrating sgRNAs. This central region colocalized with nsp3 protein, suggesting that it was an RO. Interestingly, at early time points p.i. (3 h p.i.), many RNA spots that were larger than single RNA molecules did not colocalize with nsp3, suggesting either that they were aggregates of RNA or that they represented phase-separated RNAs due to the accumulation of newly replicated RNA molecules. Because of this reason, we found that quantitating individual gRNAs or sgRNA after 2 h p.i. became challenging. More studies need to be conducted to establish the nature of these RNA spots that do not co-localize with nsp3. Our findings open avenues for further investigation into the precise nature of RNA speckles during viral replication and the molecular mechanisms governing their interaction with viral proteins. Understanding the dynamics of these early events in SARS-CoV-2 replication may offer valuable insights into the development of antiviral strategies and the elucidation of the virus’s pathogenesis.

## Figures and Tables

**Table 1:** Sequences of SARS-CoV-2 spike RNA (P1) and nsp12 RNA (P2) probes used for smRNA-FISH analyses.

Probe Number	smRNA-FISH Spike RNA Probe (P1) (5′ → 3′)	smRNA-FISH nsp12 RNA Probe (P2) (5′ → 3′)
1	tgactagagactagtggcaata	gctactttatcattgtagatgt
2	tttgtcagggtaataaacacca	agttagagaaagtgtgtctctt
3	atgtatagcatggaaccaagta	aattaccttcatcaaaatgcct
4	accatcattaaatggtaggaca	ttctacaaaatcataccagtcc
5	tatgttagacttctcagtggaa	ttttaacaaagcttggcgtaca
6	tttttgtggtaataaacaccca	agttaccattgagatcttgatt
7	gtgcaattattcgcactagaat	agaatctacaacaggaactcca
8	aataggcgtgtgcttagaatat	acatgtgactctgcagttaaag
9	gcaaatctaccaatggttctaa	ctgtcatccaaacagttaacac
10	taggttgaagataacccacata	cagcagcatacacaagtaattc
11	ctacagtgaaggatttcaacgt	gtaatagattaccagaagcagc
12	agaattccaagctataacgcag	taagtgcagctactgaaaagca
13	tataattaccaccaaccttaga	aaaattaccgggtttgacagtt
14	ggcagaaactttttgttagact	caacagaacttccttccttaaa
15	tgacaccaccaaaagaacatgg	atcctgagcaaagaagaagtgt
16	tatttgttcctggtgttataac	acgatagtagtcataatcgctg
17	gttgatctgcatgaatagcaac	ttgtctgatatcacacattgtt
18	aataaacacgccaagtaggagt	acgatgacttggttagcattaa
19	cactcatatgagttgttgacat	gaaaaccagctgatttgtctag
20	atagtgtaggcaatgatggatt	atgcgaaaagtgcatcttgatc
21	gtaagcaactgaattttctgca	tagtagggatgacattacgttt
22	agtaaaatttgtgggtatggca	tattctttgcactaatggcata
23	ggtagaatttctgtggtaacac	ctattggtcatagtactacaga
24	acttgtgcaaaaacttcttggg	cttgttccaattactacagtag
25	tggtggtgttttgtaaatttgt	tcacatttaggataatcccaac
26	aacagtaaggccgttaaacttt	acaagtgaggccataattctaa
27	agaacattctgtgtaactccaa	gaaacggtgtgacaagctacaa
28	acttgctgtggaagaaagtgag	atgaccatttcactcaatactt
29	ggagctaagttgtttaacaagc	acatacttatcggcaattttgt
30	ttgagtcacatatgtctgcaaa	cactcataaagtctgtgttgta
31	gacattttagtagcagcaagat	tcacaaagtctgtgtcaacatc
32	cctttccacaaaaatcaactct	gtcagagagtatcatcattgag
33	ctgactgagggaaggacataag	actagaccttgagatgcataag
34	agtcacatgcaagaagactaca	gaaggtacacataatcatcacc
35	gttgttgacaattcctattaca	atatcatctacaaaacagccgg
36	tgaagcattaatgccagagatg	gacacgaaccgttcaatcataa
37	gcggtcaatttctttttgaatg	tagtaagtgggtaagcatctat
38	taaattcttggcaacctcattg	atgtagctttcttatgtattgt
39	ttggagatcgatgagagattca	aatacatgtctaacatgtgtcc
40	gctataaaacctagccaaatgt	gtgtacatagcctcataaaact

## Data Availability

The data presented in this study are available on request from the corresponding author.

## Supplementary Materials

The following supporting information can be downloaded at: https://www.mdpi.com/article/10.3390/v16020262/s1, Figure S1: Presence of SARS-CoV-2 RNA within the cytoplasm and nuclei of infected cells. Vero cells infected with SARS-CoV-2 at 30 min p.i. indicating RNA in the cytoplasm (A) and in the nucleus (B). The panels represent Z-stack images of an infected cell to demonstrate the presence of the positive RNA spot inside the cell. A total of 41 Z-stacks were acquired (step size 200 nm) using wide-field microscopy starting from the bottom of a cell and moving upwards. The panel numbers in the figure represent the number of Z-stacks. The blue color represents DAPI staining and magenta represents SARS-CoV-2 spike RNA. The scale bar is 10 µm; Figure S2: A Time-course analysis of SARS-CoV-2 replication to simultaneously detect gRNA and sgRNA-S. Vero E6 cells were infected with SARS-CoV-2 and hybridized with probes at 0, 0.5, 1, 2, 3, 4, 5, and 6 h p.i. The infected cells were probed using both spike RNA probe P1 and nsp12 RNA probe P2. Four chambered slides containing infected cells and uninfected controls (mock-infected), probed with spike RNA probe P1 and nsp12 RNA probe P2, were subjected to high-speed, high-resolution scanning. The panels represent images of an entire well of infected cells at 5× magnifications. Blue represents DAPI staining, green represents gRNA detected by nsp12 RNA probe P2, magenta represents sgRNA-S detected by spike RNA probe P1, and the overlay of the two probes is shown in white. The scale bar is 500 µm; Figure S3: Representative images of SARS-CoV-2-infected Vero E6 cells demonstrating various stages of viral replication. Vero E6 cells infected with SARS-CoV-2 WA1 were subjected to RNA-FISH at 4, 5, and 6 h p.i. using nsp12 RNA probe P2, which detects gRNA. The images were acquired using HSHRS-FM. The illustration is a representative image of a field view of cells at 40× magnification for each of the three time points, showing different stages of the viral replication. The numbers in the image refer to stages 1–4 of replication, respectively. The blue color represents DAPI staining to indicate the nucleus, and the green color represents gRNA hybridized with the P2 probe. The scale bar is 20 µm.
