# Supplementary material for: Visualization of Early RNA Replication Kinetics of SARS-CoV-2 by Using Single Molecule RNA-FISH Combined with Immunofluorescence
Source: Viruses. 2024 Feb 7;16(2):262. doi: 10.3390/v16020262 (PMC10893374; doi:10.3390/v16020262)
Supplement: Supplementary file 1 [file viruses-16-00262-s001.zip › viruses-2852660-Supplementary Materials.pdf]

Supplementary Materials

Visualization of early RNA replication kinetics of SARS-CoV-2 by using single molecule RNA-FISH combined with immunofluorescence

Rajiv Pathak, Carolina Eliscovich, Ignacio Mena, Anastasija Cupic, Magdalena Rutkowska, Kartik Chandran, Rohit K. Jangra, Adolfo García-Sastre, Robert H Singer, Ganjam V. Kalpana

Supplementary Figure S1

Supplementary Figure S2

Supplementary Figure S3

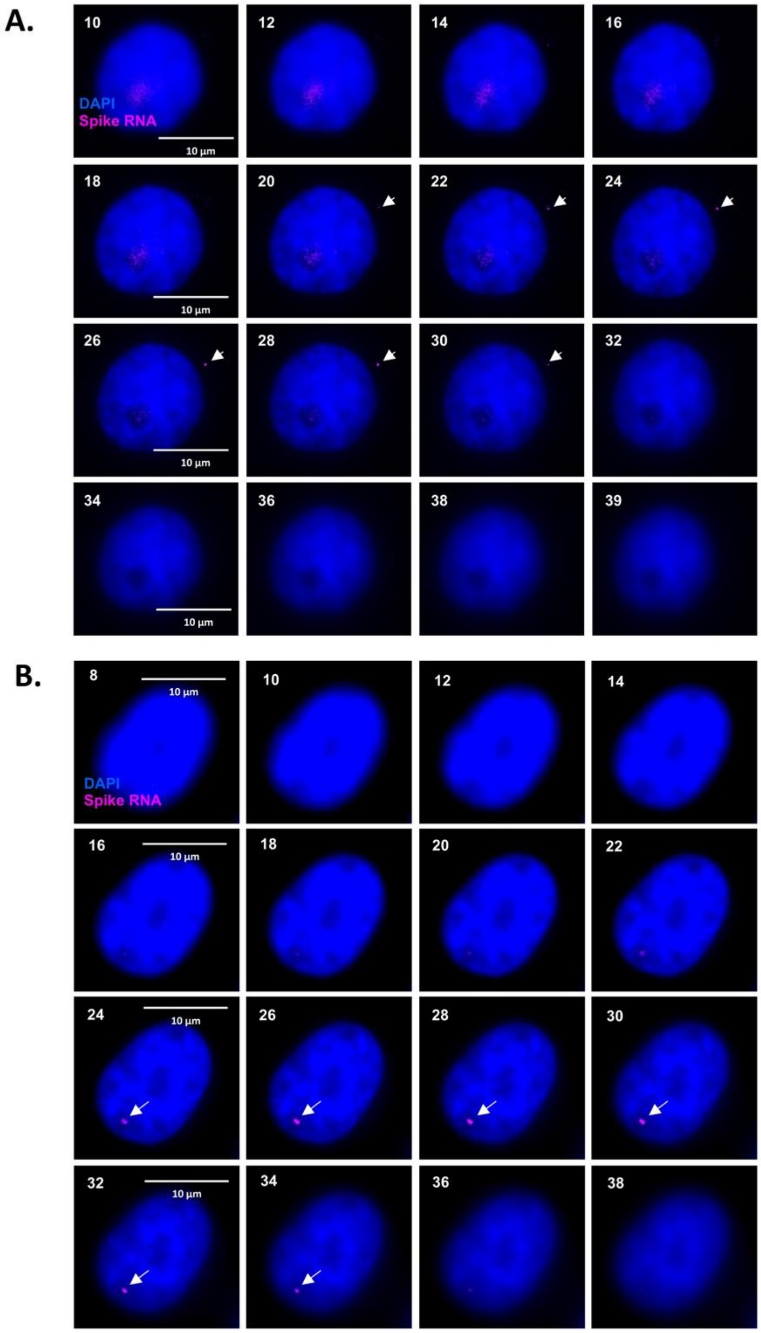

Supplementary Figure S1. Presence of SARS-CoV-2 RNA within the cytoplasm and nuclei of infected cells. Vero cells infected with SARS-CoV-2 at 30 min p.i. indicating viral RNA in the cytoplasm (A) and in the nucleus (B). The panels represent Z-stack images of an infected cell to demonstrate the presence of the positive RNA spot inside the cell. A total of 41 Z-stacks were acquired (step size 200 nm) using widefield microscopy starting from the bottom of a cell moving upwards. The panel numbers in the figure represent the stack number of the Z-stacks. Blue color represents DAPI staining and magenta represents SARS-CoV-2 spike RNA. The scale bar is 10 µm.

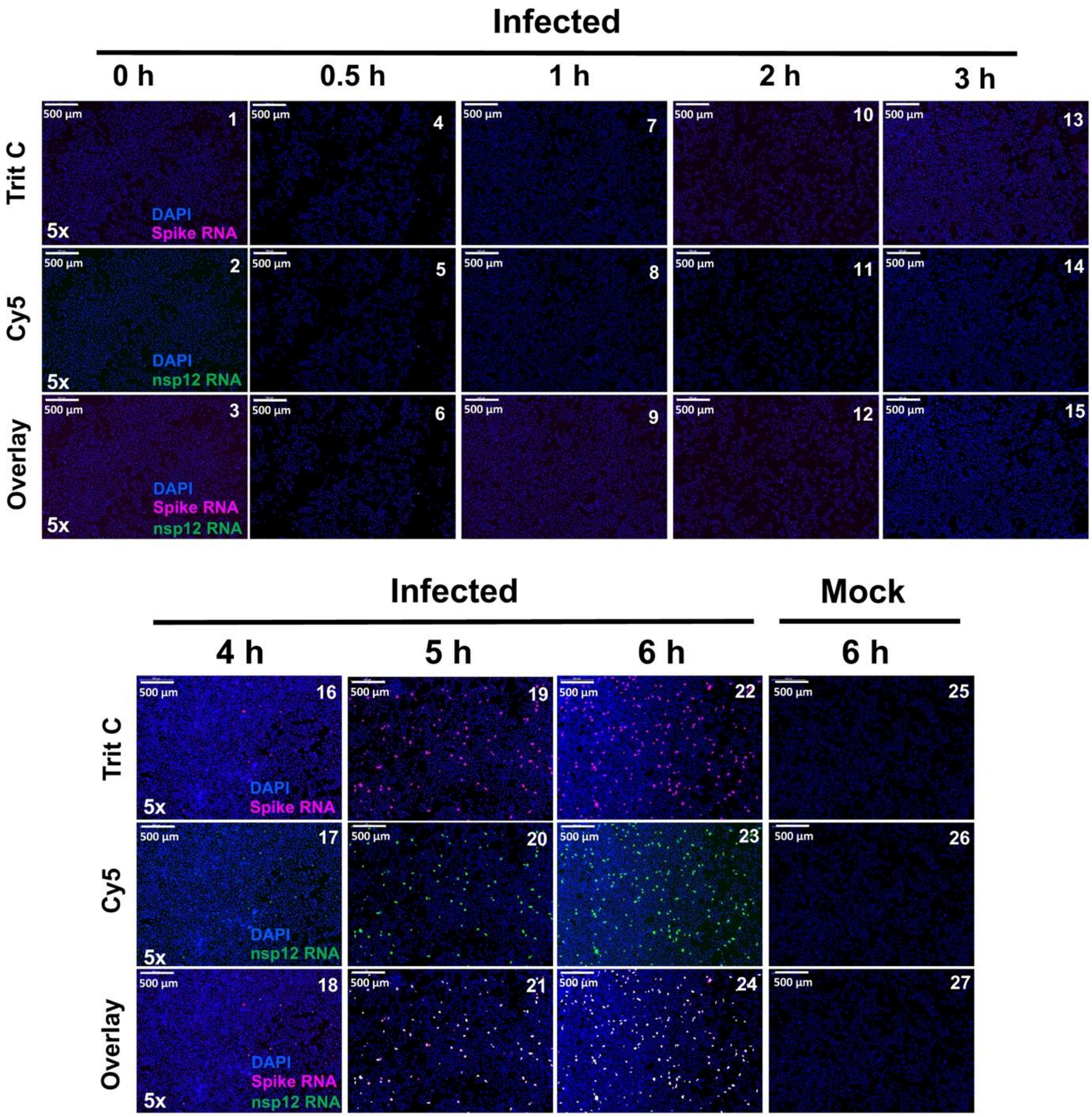

Supplementary Figure S2. A time course analysis of SARS-CoV-2 replication to simultaneously detect gRNA and sgRNA-S. Vero E6 cells were infected with SARS-CoV-2 and hybridized with probes at 0, 0.5, 1, 2, 3, 4, 5 and 6 hours p.i. The infected cells were probed using both spike RNA probe P1 and nsp12 RNA probe P2. Four chambered slides containing infected cells and uninfected controls (mock), probed with spike RNA probe P1 and nsp12 RNA probe P2, were subjected to high-speed, high-resolution scanning. The panels represent images of an entire well of the infected cells at 5x magnifications. Blue represents DAPI staining, green represents gRNA detected by nsp12 RNA probe P2, and magenta represents sgRNA-S detected by spike RNA probe P1 and the overlay of the two probes shown in white. The scale bar is 500 µm.

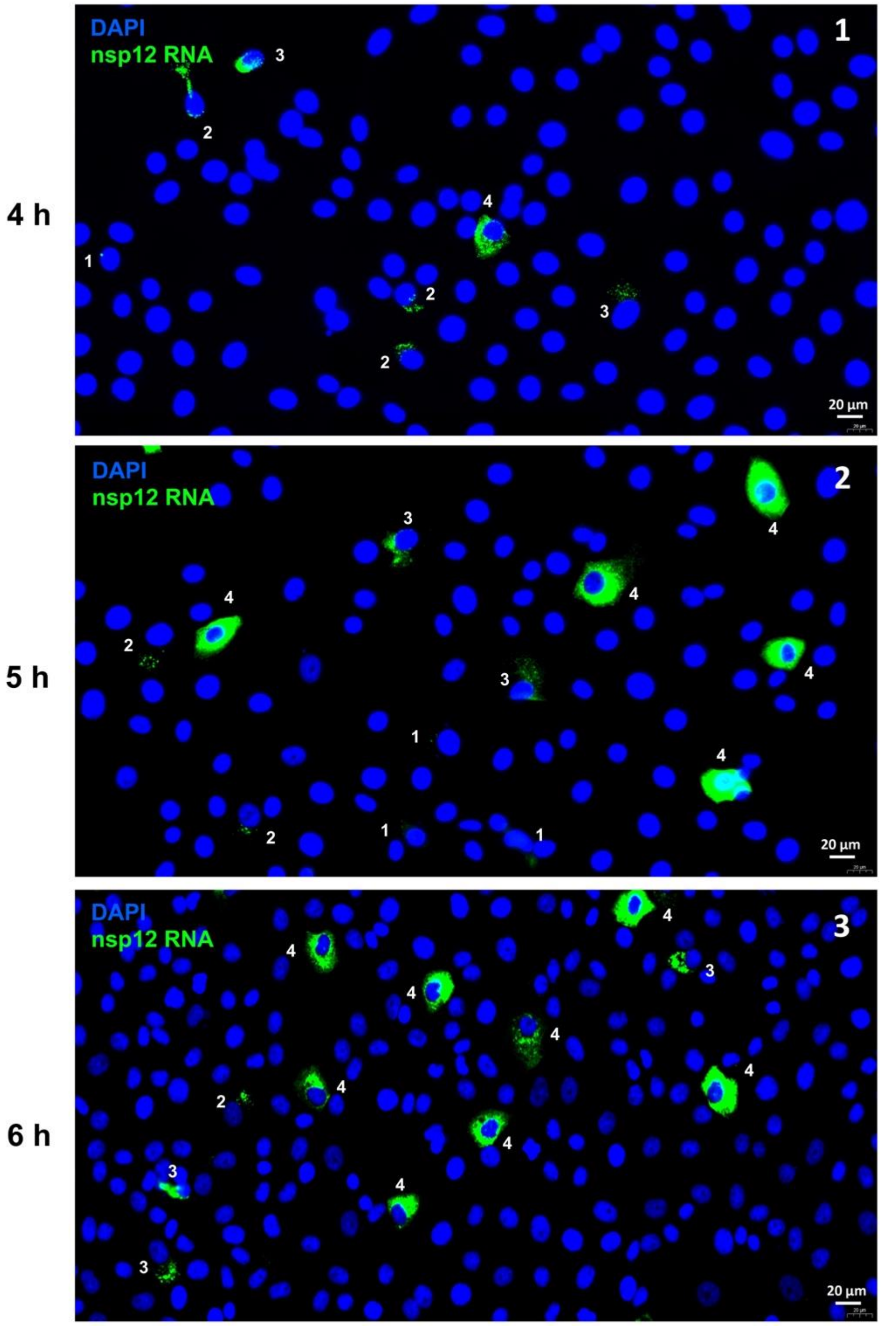

Supplementary Figure S3. Representative images of SARS-CoV-2-infected Vero E6 cells demonstrating various stages of viral replication. Vero E6 cells infected with SARS-CoV-2 WA1 were subjected to RNA-FISH at 4, 5 and 6 hours p.i. using nsp12 RNA probe P2 that detects gRNA. The images were acquired using HSHRS-FM. The illustration is a representative image of a field view of cells at 40x magnification for each of the three time-points, showing different stages of the viral replication. The numbers in the image refer to the stages of replication, stages 1-4, respectively. Blue color refers to DAPI staining to indicate the nucleus, and the green color represents gRNA hybridized with P2 probe. The scale bar is 20 µm.
